# The Impact of Implementing Operational Crisis Management Plan in Educational Hospital

**DOI:** 10.5812/ircmj.4725

**Published:** 2013-10-05

**Authors:** Mohamad Mahbobi, Shahnaze Ojaghi, Mansur Rezayi, Elahe Khorasani

**Affiliations:** 1 Kermanshah University of Medical Sciences, Kermanshah, IR Iran; 2 Isfahan University of Medical Sciences, Isfahan, IR Iran.

**Keywords:** Hospitals, Crisis Intervention, Disasters

Dear Editor,

Hospitals constitute the most important centers which accept people during disasters such as earthquakes, wars and flood. Hospitals have sophisticated organizations due to their direct contact with human health and diseases, therefore the level of service quality in such organizations is of utmost importance (1, 2). The critical conditions resulting from disasters pose great problems for hospitals. In order to enhance efficiency in critical conditions of disaster, hospitals need to have a pre-organized operational plan, lack of which will cause chaos and confusion at the time of crisis (3).

The current study was conducted in order to implement the operational plan of crisis management in Imam Reza educational hospital, and also to evaluate its effect on preparation of hospital for encountering crisis in 2009. The data collection tool was a checklist with 5 dimensions and 147 questions. Validity of the questionnaire was confirmed with the viewpoints of university professors, managers of hospitals who involved in crisis, experts in crisis management of the Red Crescent organization of Kermanshah Province. The reliability was calculated Cronbach's α = 0.800. Initially, training classes of crisis management were held for all managers, authorities and personnel. Subsequently, the organizational chart of hospital crisis management was designed and implemented, the human resources were organized according to the chart, and ultimately, maneuvers and drills of crisis preparation were conducted.

The findings indicate that the level of preparation of the information system for encountering crisis improved from 61.8% before intervention to 90.9% after. The level of preparation of the command center for encountering crisis in Imam Reza hospital improved from 74.1% to 90.3% following the intervention.

Training for encountering crisis improved from 58.3% before intervention to 91.8% after intervention. The intervention improved the organization of personnel for encountering crisis from 75% to 93.5%. Prior to intervention, no maneuvers had been conducted in the hospital in the case of crisis; the intervention improved this figure from 0% to 93.3%. As a result, general preparation of the hospital for a coming crisis improved from 53.8% to 92%.

The intervention improved the information system of Imam Reza Hospital from 61.7% to 90.9%. A study conducted by Mosadegh Rad et al. in Isfahan reported the status of information and communicative system of hospitals to be 32% (4). The crisis command center vigilance of hospital improved from 61.7% before intervention to 90.3% after intervention. Nowadays, command center constitutes the most common system of crisis management worldwide, and its acceptance is increasing due to its successful results. A study by Mahbubi reported that the crisis command centers were feeble which is similar to our pre-intervention findings (5).

The level of preparation of organization in Imam Reza hospital improved from 75% before intervention to 93.5% after. One study concerning the same issue in educational hospitals of Isfahan reported that the level of preparation was 54% (6) while another finding was not in accordance with Imam Reza hospital and showed greater preparation for organization.

In fact, it is essential to organize a crisis management system in hospitals consisting of specified posts in organizational chart, in which each post will accomplish a certain mission under critical conditions. Such a system requires a logical structure of management, clarifying the responsibilities and creating canals for unambiguous reports. In general, the level of preparation for crisis in Imam Reza educational hospital in Kermanshah was 53% before intervention and improved to 92%. Crisis management has been implemented in Amir-ol-Momenin hospital in Tehran with a command center-based organizational chart determining the responsibilities of respective individuals, which is similar to our study (7).

The findings indicate that the implementation of hospital crisis management system in Imam Reza hospital of Kermanshah university of medical sciences improved its preparation to an acceptable level.

In conclusion, we recommend organizing crisis management centers in Iranian hospitals in order to prepare operational plans for encountering crises based on the unique features of each respective hospital. Such plans need to be organized in manuals and executive instructions.
